# PDGF upregulates CLEC-2 to induce T regulatory cells

**DOI:** 10.18632/oncotarget.5765

**Published:** 2015-09-21

**Authors:** Sudhanshu Agrawal, Sreerupa Ganguly, Pega Hajian, Jia-Ning Cao, Anshu Agrawal

**Affiliations:** ^1^ Division of Basic and Clinical Immunology, Department of Medicine, University of California, Irvine, CA, USA

**Keywords:** Clec-2, dendritic cell, platelets, T regulatory cells, PDGF, podoplanin, Immunology and Microbiology Section, Immune response, Immunity

## Abstract

The effect of platelet derived growth factor (PDGF) on immune cells is not elucidated. Here, we demonstrate PDGF inhibited the maturation of human DCs and induced IL-10 secretion. Culture of PDGF-DCs with T cells induced the polarization of T cells towards FoxP3 expressing T regulatory cells that secreted IL-10. Gene expression studies revealed that PDGF induced the expression of C-type lectin like receptor member 2, (CLEC-2) receptor on DCs. Furthermore, DCs transfected with CLEC-2 induced T regulatory cells in DC-T cell co-culture. CLEC-2 is naturally expressed on platelets. Therefore, to confirm whether CLEC-2 is responsible for inducing the T regulatory cells, T cells were cultured with either CLEC-2 expressing platelets or soluble CLEC-2. Both conditions resulted in the induction of regulatory T cells. The generation of T regulatory cells was probably due to the binding of CLEC-2 with its ligand podoplanin on T cells, since crosslinking of podoplanin on the T cells also resulted in the induction of T regulatory cells. These data demonstrate that PDGF upregulates the expression of CLEC-2 on cells to induce T regulatory cells.

## INTRODUCTION

Inhibition of immune response is essential to prevent uncontrolled cell activation and inflammation, both of which are detrimental to the body. Chronic inflammation is the hallmark of autoimmune diseases such as rheumatoid arthritis, multiple sclerosis etc [1, 2]. Inflammation is controlled by regulatory T cells (Tregs) which are potent suppressors of cellular activation. It is well established that the number and function of Tregs is severely compromised in inflammatory and autoimmune disorders [1, 3, 4]. Immunotherapy with Tregs is also useful in transplantation where these cells can dampen the immune response and prevent graft rejection [5].

Antigen presenting cells such as dendritic cells (DCs) are capable of inducing Tregs. DCs play a crucial role in initiating immunity against invading pathogens and maintaining tolerance against self-antigens [6–8]. Encounter with pathogens activates DCs resulting in upregulation of antigen presenting and costimulatory molecules along with secretion of pro-inflammatory cytokines. In contrast, self-antigens induce secretion of anti-inflammatory cytokines [6–8]. Absence of costimulation and secretion of anti-inflammatory cytokines results in DCs which induce T cell tolerance. Such DCs are known as tolerogenic DCs (Tol DC). Tol DCs induce T cell tolerance by generation of Ag-specific, anergic CD4^+^ T cells and the peripheral induction of CD4^+^CD25^+^Foxp3^+^ T regulatory cells (Tregs) [7, 9].

This has led to the development of a number of *in vitro* methods for the generation of Tol DCs. Both genetic and pharmacological inhibitors have been investigated. Modification of DC *in vitro* with immunosuppressive cytokines such as IL-10, transforming growth factor-β (TGF-beta) or molecules such as indoleamine dioxygenase [IDO], is a relatively simple way to generate TolDC [10, 11]. Similarly, *in vitro* treatment of DCs by molecules that prevent their activation also generates Tol DCs. Examples include drugs that inhibit nuclear factor κB (NFκB) signaling-for example, the BAY 11-7085 compound, dexamethasone and vitamin D_3_ [12, 13].

Platelets release several factors such as, TGF-β, vascular endothelial growth factor (VEGF), and platelet-derived growth factor (PDGF) on aggregation [14–16]. PDGF along with VEGF is considered a key driver of angiogenesis [17]. PDGF and its receptor, platelet-derived growth factor receptor-β (PDGFR-β), are essential to pericyte recruitment, a critical component of maturing blood vessels [17, 18]. In addition to their effects on vasculature, these factors also affect the immune cells since the receptors for these factors are also expressed on DCs and T cells [19]. Both TGF-β and VEGF have been demonstrated to suppress DC activation [20, 21]; however, the effect of PDGF on DCs has not been investigated.

Here we report that PDGF has a profound effect on human DC functions and induces T regulatory cells via the expression of C-type lectin like receptor member 2 (CLEC-2).

## RESULTS

### PDGF induces IL-10 in DCs

**Figure 1:**
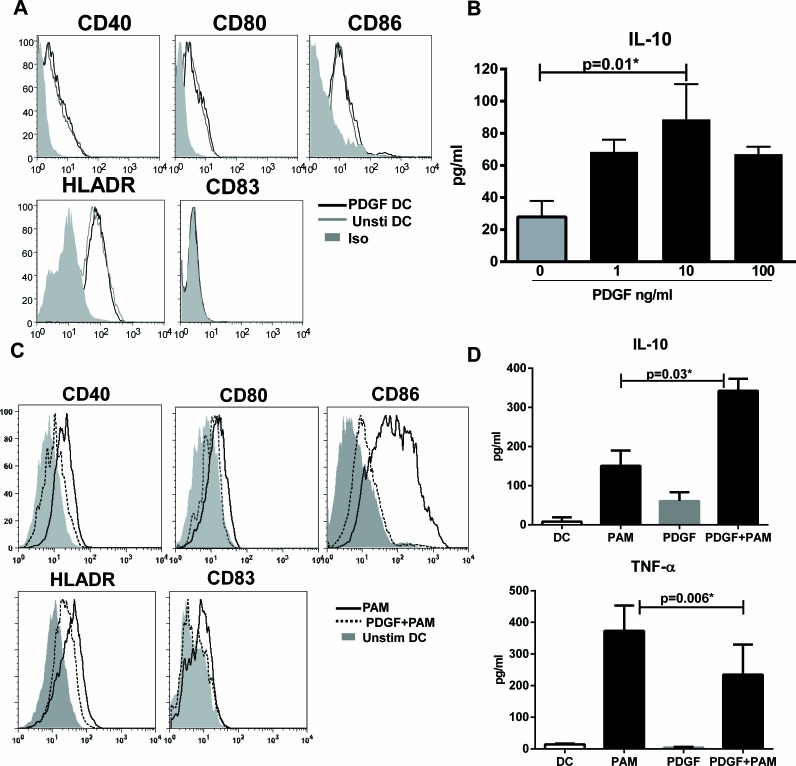
PDGF induces IL-10 in DCs DCs were cultured with PDGF AB at (1-100ng/ml) for 48h. **A.** Histograms depict the expression of costimulatory and antigen presenting molecules on PDGF stimulated DC (PDGF-DC) and unstimulated DC. Data is representative of 6 such experiments. **B.** Bar graph depicts the level of IL-10 secreted by PDGF-DC and unstimulated DC. DCs were exposed to PDGF for 24h and subsequently stimulated overnight with PAM. **C.** Histograms depict the expression of costimulatory and antigen presenting molecules on PAM stimulated DC (PAM), PDGF exposed +PAM-stimulated DCs (PDGF+PAM) and unstimulated DC. Data is representative of 6 such experiments. **B.** Bar graph depicts the level of IL-10 secreted by PDGF-DC and unstimulated DC. Data is mean +/− S.E. of 4 different subjects.

PDGF exists as 3 different isoforms in humans, PDGF-AA, PDGF-BB and PDGF-AB with PDGF-AB being the most abundant isoform [17, 22]. DCs were cultured with PDGF-AB at concentrations ranging from 1-100ng/ml for 48h. Addition of PDGF did not lead to change in expression of antigen presenting (HLADR) and maturation makers (CD40, CD80, CD86, CD83) on DCs (Figure 1A). Data presented is with PDGF at 10ng/ml since other concentrations of PDGF were comparable.

The cytokine secretion by DCs was determined using multiplex bead assay. PDGF stimulated DC (PDGF-DC) secreted significantly higher (*p* < 0.05) levels of IL-10 compared to unstimulated DCs (Figure 1B). The secretion followed a bell shaped curve with maximum secretion being observed at a concentration of PDGF 10ng/ml. The levels of TNF-α, CXCL-8, IL-6, MCP-1, and CXCL-10 were comparable to unstimulated DCs (data not shown). These data suggest that DCs stimulated with PDGF may be immunosuppressive.

To further confirm that indeed PDGF is immunosuppressive, DCs were treated with PDGF and subsequently stimulated with TLR-2 ligand, PAM-3 Cysteine (PAM). As is evident from Figure 1C, exposure to PDGF inhibited the upregulation of DC maturation markers by PAM. Moreover, the secretion of pro-inflammatory cytokine, TNF-α was significantly reduced (*p* < 0.05) while the secretion of IL-10 was significantly increased (*p* < 0.05) in PDGF exposed, PAM-stimulated DCs (PDGF-PAM) (Figure 1D). Together, these data suggest that exposure to PDGF renders the DCs tolerogenic.

### PDGF stimulated DCs induce T regulatory cells

**Figure 2:**
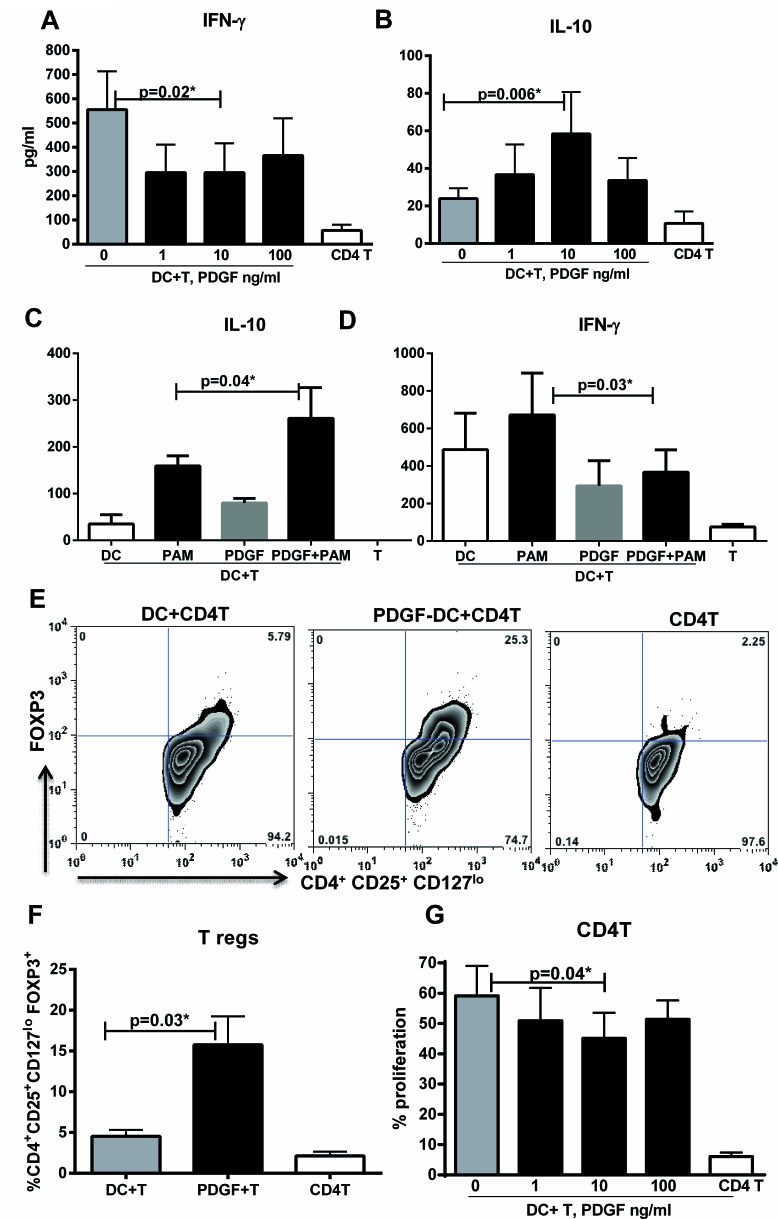
PDGF-DC induces T regulatory cells PDGF-DC and unstimulated DC were cultured with CD4T cells for 5 days. Bar graphs depict the levels of **A.** IL-10 and **B.** IFN-γ secreted by T cells. Data is mean +/− S.E. of 5 different subjects. PDGF-DC, PAM-stimulated DCs, PDGF-PAM DCs and unstimulated DC were cultured with CD4T cells for 5 days. Bar graphs depict the levels of **C.** IL-10 and **D.** IFN-γ secreted by T cells. Data is mean +/− S.E. of 4 different subjects. **E.** Zebra plot depicts the percentage of FoxP3^+^ cells in gated CD4^+^CD25^+^CD127^lo^ cells in DC-T cell coculture. Figure is representative of 4 such experiments. F. Bar graph depicts the mean +/− S.E. of the same. **E.** Bar graph depicts the percent of T cell proliferation as measured by CFSE dye dilution. Data is mean +/− S.E. of 6 different subjects.

We then investigated whether increased IL-10 secretion by DCs resulted in polarization of T helper cell response towards a T regulatory phenotype. PDGF-DC and unstimulated DCs were cultured with purified, CFSE labeled naïve CD4 T cells for six days. IFN-γ secretion from PDGF-DC-T co-culture was significantly (*p* < 0.05) lower and IL-10 secretion was significantly (*p* < 0.05) higher than that of unstimulated DC-T co-culture (Figure 2A & 2B). The level of TGF-β in the supernatant was below the limit of detection (data not shown). IL-4, IL-5 levels were also comparable (data not shown). Similar results were obtained with T cells cultured with PDGF-treated PAM stimulated DCs (Figure 2C & 2D). Staining of T cells with FoxP3, signature regulatory T cell transcription factor revealed that PDGF stimulated DC significantly enhanced the differentiation of T helper cells towards Tregs verses the unstimulated DC (Figure 2E & 2F). The proliferation of CD4 cells was also significantly (*p* < 0.05) reduced after culture with PDGF-DCs as compared to unstimulated DCs (Figure 2G). PDGF stimulated DC thus bias the T cell response towards a regulatory phenotype.

### PDGF upregulates the expression of C-type lectin receptor, CLEC-2 on DCs

**Figure 3:**
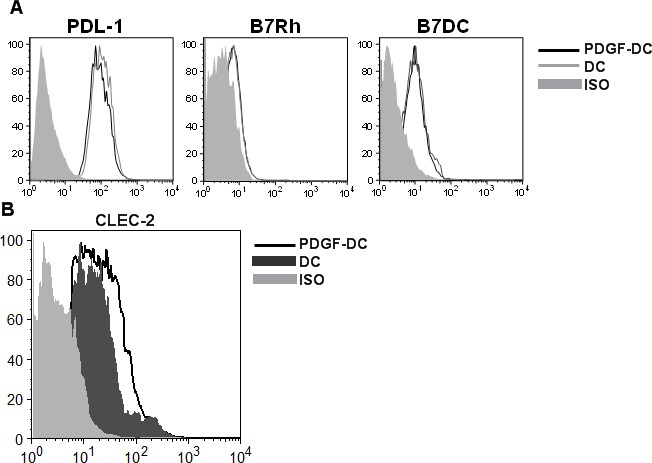
PDGF upregulates the expression of C-type lectin receptor, CLEC-2 on DCs DCs were cultured with PDGF AB at (1-100ng/ml) for 48h. Histograms depict- **A.** the expression of inhibitory costimulatory molecules on DCs. **B.** The expression of CLEC-2 on DCs. Data is representative of 6 such experiments.

Expression of a variety of inhibitory costimulatory molecules on DCs can induce T regulatory cells [12]. Here we determined the expression of Programmed Cell death Ligand 1 (PDL-1), PDL-2 (B7DC) and B7Rh on PDGF-DCs using specific antibodies. There was no significant difference in the expression of these molecules between PDGF stimulated DC and controls (Figure 3A). Since expression of numerous other inhibitory costimulatory molecules as well as other molecules such as IDO in DCs can induce the differentiation of T regulatory cells, we performed affymetrix gene expression microarray to determine the difference between PDGF stimulated DC and unstimulated DC. The gene expression pattern of both groups was highly homologous and only 12 (all upregulated) genes were significantly different between the two groups (Table 1). None of the genes were known to induce T regulatory cells. One of gene which was significantly upregulated on PDGF stimulated DC was CLEC-2 which is C-type lectin receptor expressed primarily on platelets [23]. Since CLEC-2 was the only gene which was expressed on cell surface and may possibly interact with T cells to generate Tregs we focused our attention on this gene. The expression of CLEC-2 receptor was compared between PDGF-DC and unstimulated DC using CLEC-2 specific antibodies (Figure 3B). The expression was significantly higher (*p* < 0.05) on PDGF-DCs. These data demonstrate that PDGF induces the expression of CLEC-2 on DCs.

**Table 1:** A list of 12 genes with expression changes in DCs activated with PDGF and meeting the criteria of *p* < 0.05 (student's t-test), FDR < 5%, and fold change ≥ 1.25

Gene Identifier	Other ID	Gene ID	Ratio	Direction	*p*-value
NM_053039	8095404	UGT2B28	1.39	Up	0.023
NM_005267	7904927	GJA8	1.37	Up	0.044
NM_021176	8046116	G6PC2	1.33	Up	0.004
NM_001130711	7961091	CLEC2A	1.32	Up	0.028
NM_001104	7941662	ACTN3	1.27	Up	0.037
NM_175057	8122127	TAAR9	1.26	Up	0.010
NM_181538	8141371	GJC3	1.26	Up	0.013
NM_003081	8060963	SNAP25	1.26	Up	0.004
NM_001753	8135594	CAV1	1.25	Up	0.044
NM_001105578	8034583	SYCE2	1.25	Up	0.010
NM_001003395	8121838	TPD52L1	1.25	Up	0.001

### CLEC-2 expression on DC induces T regulatory cells

**Figure 4:**
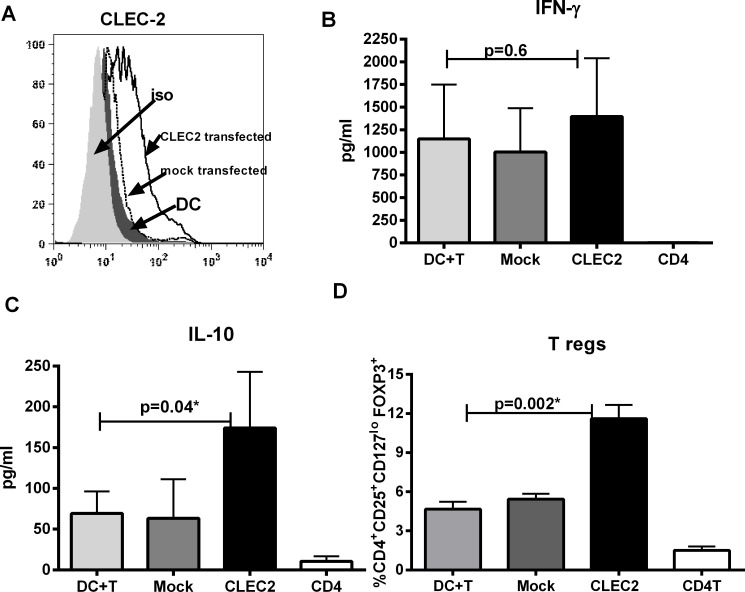
CLEC-2 expression on DC induces T regulatory cells DCs were transfected with CLEC-2 and subsequently cultured with T cells for 5 days. **A.** Histograms depict the expression of CLEC-2 on DCs 48h after transfection. Mock is mock transfected DC. Data is representative of 5 such experiments. Bar diagram depicts the levels of **B.** IFN-γ and **C.** IL-10 and secreted by T cells. Data is mean +/− S.E. of 6 different subjects. **C.** Bar graph depicts depicts the percentage of FoxP3^+^ cells in gated CD4^+^CD25^+^CD127^lo^ cells in DC-T cell coculture. Data is mean +/− S.E. of 6 subjects.

To confirm that expression of CLEC-2 was responsible for regulatory nature of PDGF-DC, unstimulated DCs were transfected with CLEC-2 plasmid (gift from Dr. Steve Watson, University of Birmingham, U.K.)[24] using magnetofection. This resulted in significant expression of CLEC-2 on DCs 48h after transfection (Figure 4A). CLEC-2 expressing DCs were collected and cultured with T cells as described in Figure 2. Determination of cytokines in the supernatant revealed that while the IFN-γ levels were comparable (Figure 4B), IL-10 secretion was significantly increased in CLEC-2 DC-T co culture (Figure 4C). Moreover, culture of CLEC-2 transfected DCs with T cells generated significantly increased percent of Treg cells as compared to mock transfected DCs (Figure 4D). Thus CLEC-2 expression in DCs bestows the ability to induce the differentiation of Tregs.

### CLEC-2 expression on platelets also induces T regulatory cells

**Figure 5:**
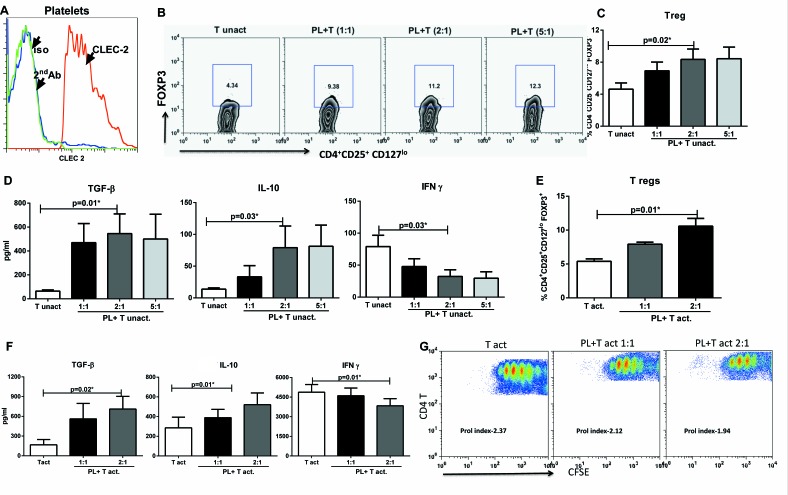
CLEC-2 expression on platelets induces T regulatory cells Platelets were cultured with T cells for 96h. **A.** Histogram depicts the expression of CLEC-2 on freshly isolated platelets. **B.** Zebra plot depicts the percentage of FoxP3^+^ cells in gated CD4^+^CD25^+^CD127^lo^FoxP3^+^ cells in PL-CD4+T (unactivated) cell coculture. Figure is representative of 5 such experiments. **C.** Bar graph depicts the mean +/− S.E. of the same. **D.** Bar graphs depict the production of TGF-β, IL-10 and IFN-γ in the co-culture. Data is mean +/− S.E. of 5 different subjects. **E.** Bar graph depicts the percentage of FoxP3^+^ cells in gated CD4^+^CD25^+^CD127^lo^FoxP3^+^ cells in PL-T (anti CD3+anti- CD28 activated) cell coculture. **F.** Bar graphs depict the production of TGF-β, IL-10 and IFN-γ in the co-culture. Data is mean +/− S.E. of 5 different subjects. **G.** Dot plots depict the T cell proliferation in PL- T (act) coculture as measured by CFSE dye dilution. Prol index is proliferation index. Figure is representative of 4 such experiments.

Next we determined if CLEC-2 expression on APCs such as DCs is required for inducing T regs or any cell expressing CLEC-2 is capable of inducing the T regs. Platelets in the blood naturally express CLEC-2 to interact with endothelium [23, 25]. Therefore, we purified platelets from the blood and confirmed the expression of CLEC-2 on purified platelets (Figure 5A). The purified platelets were cultured with syngeneic unactivated T cells and the induction of T regs was determined. As shown in Figure 5B, culture of platelets with T cells resulted in significant increase (*p* < 0.05) in CD4^+^CD25^+^CD127^lo^ FoxP3^+^, Tregs (Figure 5B & 5C). This increase was proportional to the number of platelets. Platelet to T cell ratio of 2:1 was found to be optimal since the increasing the ratio to 5:1 did not result in significant increase over 2:1. T cells cultured with Platelets also secreted high levels of TGF-β, IL-10 compared to T cell cultured alone (Figure 5D). The secretion of IFN-γ was concomitantly reduced. Culture of platelet alone did not result in detectable level of these cytokines (data not shown).

Next we determined the effect of platelets on anti-CD3 and anti-CD28 bead activated T cells. Treg induction (Figure 5E) and cytokine secretion (Figure 5F) results were similar to that obtained with unactivated T cells. In addition, we also measured proliferation. The proliferation index of T cells was also significantly reduced (*p* < 0.05) in the presence of platelets (Figure 5G).

Collectively, these results suggest that CLEC-2 expressing cells stimulate T cells to induce a T regulatory phenotype.

### Addition of soluble CLEC-2 protein/ podoplanin antibody to T cells also induces T regulatory cells

**Figure 6:**
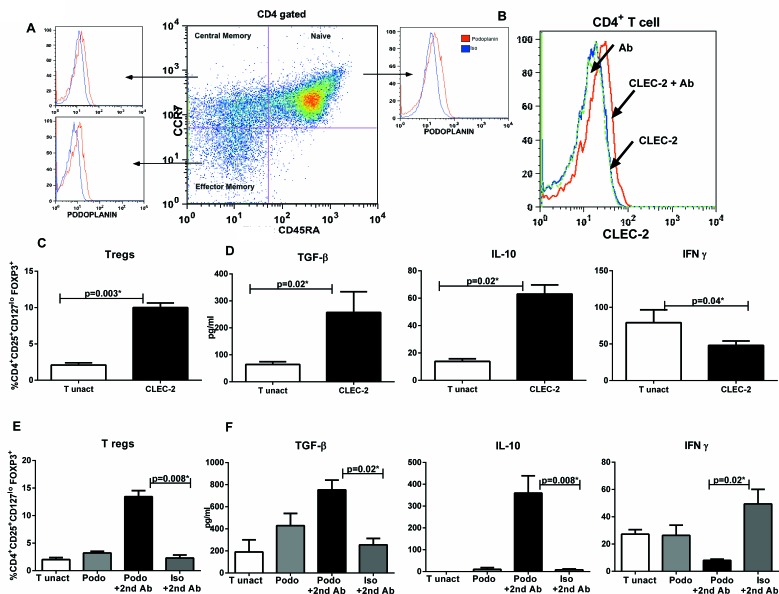
Addition of CLEC-2 protein/podoplanin antibody to T cells induces T regulatory cells **A.** Histograms depict the expression of Podoplanin of on naïve, central memory and effector memory CD4 T cells. Figure is representative of 3 such experiments. **B.** Histogram depicts the binding of CLEC-2 protein on freshly isolated CD4 T cells. Figure is representative of 3 such experiments. Purified T cells were cultured with CLEC-2 protein for 96h. **C.** Bar graph depicts the percentage of FoxP3^+^ cells in gated CD4^+^CD25^+^CD127^lo^FoxP3^+^ cells in CD4+T cells cultured with CLEC-2. **D.** Bar graphs depict the production of TGF-β, IL-10 and IFN-γ by T cells cultured in the presence of CLEC-2 protein. Data is mean +/− S.E. of 4 different subjects. Purified T cells were cultured with either podoplanin antibod (podo) or podo+2^nd^ antibody or isotype antibody (iso) + 2^nd^ antibody for 96h. C. Bar graph depicts the percentage of FoxP3^+^ cells in gated CD4^+^CD25^+^CD127^lo^FoxP3^+^ cells. D. Bar graphs depict the production of TGF-β, IL-10 and IFN-γ by T cells. Data is mean +/− S.E. of 4 different subjects.

The two known ligands for CLEC-2 are the Podoplanin and snake venom rhodotoxin [23, 26]. Recent studies have demonstrated that T cells express Podoplanin in mice [27]. We confirmed the expression of Podoplanin on human CD4 T cells (Figure 6A). Podoplanin was expressed on naïve, central and effector memory subsets with highest expression on effector memory subset. Next, we determined whether purified, soluble CLEC-2 protein can bind to T cells. Indeed, there was significant binding of CLEC-2 on T cells (Figure 6B). Since CLEC-2 expression on two very different cell types, DCs and platelets was leading to the induction of Tregs, we investigated if the protein itself was enough to generate Tregs or it requires accessory signals from the cells. Purified T cells were cultured with recombinant CLEC-2 and induction of Tregs was determined. Addition of CLEC-2 alone resulted in increase in Tregs (Figure 6C). The production of TGF-β and IL-10 was also significantly higher (*p* < 0.05) in the T cells cultured with CLEC-2 protein (Figure 6D). IFN-γ secretion was very low; despite this, it was reduced in CLEC-2 treated T cells (Figure 6D). These data demonstrate that CLEC-2 protein by itself is sufficient to induce Tregs.

Since CLEC-2 most likely interacted with podoplanin on T cells, we determined whether crosslinking of podoplanin on T cells using specific antibody also resulted in induction of T regs. Indeed, as shown in Figure 6E, ligation of podoplanin on unactivated T cells resulted in significantly increased (*p* < 0.05) induction of T regs compared to T cells crosslinked with isotype specific antibody. Similar to CLEC-2, podoplanin antibody treatment of T cells also resulted in significantly increased secretion of TGF-β and IL-10 with a concomitant reduction IFN-γ Figure 6F).

Taken together, these results demonstrate that CLEC-2 protein binds with podoplanin on T cells to induce T reg cells.

## DISCUSSION

Here we demonstrate that C-type lectin receptor; CLEC-2 induces Tregs in soluble form as well as when expressed on DCs and platelets.

CLEC-2 belongs to the family of C-type lectin receptors (CLRs) which are a large family of carbohydrate binding, Ca^++^ dependent receptors present on numerous cells types [28]. CLEC-2 is a member of the “dectin-1 cluster” of CLRs and was originally thought to be restricted to platelets. Recent studies document the expression of the receptors in the ‘Dectin-1 cluster’ on myeloid populations where they function in homeostasis and immunity [28–32]. Initial studies suggested that surface expression of CLEC-2 is restricted to platelets, although RT-PCR analysis has shown transcripts in PBMC, bone marrow cells, monocytes, dendritic cells and granulocytes [25, 33]. However, recent studies have reported the expression of CLEC-2 on DCs in inflammatory conditions [34, 35] where based on our observations, CLEC-2 may have a role in suppressing inflammation.

Podoplanin, a sialoglycoprotein is the physiological ligand for CLEC-2 [23, 25]. It is expressed on numerous cell types including mesothelial cells, epithelial cells, fibroblasts, follicular dendritic cells and various tumor cells. In tumors, podoplanin is involved in tumor cell-induced platelet aggregation and tumor metastasis [36]. Podoplanin is also expressed at high levels on the endothelial cells and fibroblastic reticular cells of lymph nodes where it plays a major in maintaining the high endothelial venule integrity during immune response via its binding with CLEC-2 on platelets[37]. Podoplanin-CLEC-2 axis also controls the expansion and contraction of lymph nodes during an immune response [30, 38]. CLEC-2 expression on DCs and its interaction with podoplanin in fibroblastic reticular cells was also found to be essential for migration of DCs to lymphatics and trafficking within lymph nodes [29]. All these studies have studied the interaction of CLEC-2 on DCs with podoplanin on fibroblast reticular cells. There is a key gap in knowledge about the effect of CLEC-2 expressing DCs on T cells. Peters et al [27] recently demonstrated that podoplanin is expressed on effector T cells during autoimmune inflammation where it functions as an inhibitory molecule promoting tolerance. Our results also demonstrate that CLEC-2 binds to T cells (Figure 6B) and ligation of podoplanin on T cells induces T reg cells (Figure 6E–6F). Therefore, CLEC-2 may be inducing Tregs via engagement of podoplanin on T cells.

Our discovery that PDGF can induce regulatory DCs identifies novel approaches to generate regulatory or tolerogenic DCs. Furthermore, our finding that CLEC-2 protein by itself and podoplanin antibody are capable of inducing T regs identifies novel approaches for induction of Tregs for immunotherapy against transplantation and autoimmunity. Local infusion of CLEC-2 or podoplanin antibody in autoimmune diseases such as Multiple Sclerosis, rheumatoid arthritis may prove useful in suppressing inflammation. This is supported by the fact that global deletion of podoplanin in mice resulted in enhanced T cell responses as well as spontaneous development of EAE [27]. Furthermore, podoplanin expressed on Th17 cells in mice [27, 39, 40] inhibits the long-term Th17 responses in the target organ by reducing sensitivity to IL-7 survival signals, thereby preventing tissue damage.

Our results also suggest a novel mechanism by which platelets may be inducing immune suppression and promoting tumor growth. The activation of platelets releases PDGF which not only causes platelet aggregation and angiogenesis but also induces tolerance in DCs via induction of CLEC-2. PDGF is also known to upregulate the expression of CLEC-2 on platelets. Both these cells together enhance the induction of Tregs in the tumor microenvironment. Our observations suggest novel targets for treatment of tumors. Inhibitors of CLEC-2 which prevent the binding of of CLEC-2 with podoplanin on T cells may prove useful in reducing immunosuppression in tumor microenvironment. However, such therapies would require careful design so that the effect is specific to tumors and does not induce other physiological interactions of CLEC-2. In this regard, Kato et al have recently reported the development of a podoplanin antibody which specifically recognizes podoplanin on tumor cells and not on endothelial cells [41].

To our knowledge this the first report of CLEC-2 expression of DCs and its binding with podoplanin on T cells in humans. As mentioned, this knowledge has the potential to lead to the development of novel therapies against tumors and autoimmunity.

## MATERIALS AND METHODS

### Blood donors

Blood samples were obtained from healthy volunteers via Institute for Clinical and Translational Science (ICTS), UC Irvine. This study was approved by the Institutional Review Board of the University of California (Irvine, CA).

### Culture and stimulation of human monocyte-derived DCs

Monocyte derived DCs were prepared as described before by culturing the purified monocytes with GMCSF and IL-4. DCs (CD14^−^HLA-DR^+^CD11c^+^ cells) were collected after 6 days [32]. Serum free, AIM V media was used for culture. The purity of the DCs was >95% as determined by the expression of CD14, CD11c and HLA-DR. DCs collected were stimulated with recombinant PDGF-AB (Peprotech, NJ) at a concentration ranging from 1-100ng/ml. 48h later supernatant was collected and stored at −70°C until analyzed. In experiments with PAM, DCs were treated with PDGF for 24h and subsequently stimulated overnight with PAM 3 Cys (Invivogen) at a concentration of 10μg/ml. Cytokines, IL-6, IL-10, TNF-α, CXCL-8, MCP-1, CXCL-10 in the supernatants was measured by multiplex cytokine assay (eBiosciences, CA) as per the manufacturer's protocol.

Control and PDGF-stimulated DCs (PDGF-DC) were analyzed for the expression of CD40, CD80, CD86, CD83, PDL-1, B7DC, B7Rh and HLADR (BD Bioscience) using Flow jo (Treestar Inc).

### Allogeneic DC-T cell co-cultures

PDGF-stimulated and unstimulated DCs were cultured with magnetic bead purified (StemCell, Vancouver, Canada), allogeneic T cells at a ratio of 1:10. After 6 days of incubation, the supernatant was collected and the secretion of TGF-β, IL-5, IL-4, IFN-γ, IL-10, (BD Bioscience) was assessed using ELISA. Subsequently, the cells were collected and stained with antibodies for CD4, CD25, CD127 and FoxP3 to stain for Tregs. CD4^+^CD25^+^CD127^lo^ gated cells were analyzed for the expression of FoxP3. In some assays to measure proliferation, T cells were labeled with CFSE and proliferation was measured by flow cytometry as described [42].

### Genearray of DCs

DCs and PDGF-DCs were generated as described above. RNA was extracted from DC and corresponding PDGF-DC from 3 different subjects using TRI reagent as in the past [43]. Affymetrix gene array (Human gene 1.0ST array) was performed by UCI genomics high throughput facility as per the manufacturer's instructions. Genearray data was analyzed by Genesifter.

### CLEC-2 transfection and staining

CLEC-2 plasmid was a kind gift from Dr. Steve Watson U.K [24]. CLEC-2 was transfected into immature DCs using CombiMag Magnetofection (Chemicell, Berlin, Germany) kit as per the manufacturer's instructions. Magnetofection™ is a novel, simple and highly efficient method to transfect cells in culture. It exploits magnetic force exerted upon gene vectors associated with magnetic particles to draw the vectors into the target cells. In this manner, the full vector dose applied gets concentrated on the cells within a few minutes so that 100% of the cells get in contact with a significant vector dose. Briefly, immature DCs were derived from monocytes. 5×10^5^ DCs were added/ well of a 24 well plate. Non-adherent cell protocol for CombiMag was used. Transfection reagent was Roche XtremeGENE.

Expression of CLEC-2 was determined 48h after transfection using specific antibody (RnD systems) followed by FITC conjugated second antibody.

### Platelet and T cell co-cultures

Platelets were purified from whole blood using the protocol described [44]. Briefly blood was centrifuged at 200 x g or 15min at room temperature to obtain platelet-rich plasma. To obtain a platelet pellet, the collected plasma was centrifuged at 250 x g for 5 min at RT. The pellet was suspended in AIM V. T cells were purified from the same donor using magnetic beads as described above. Platelets and T cells were cultured at ratios ranging from 1:1 to 1:5 for 72h. Subsequently, the cells were collected and stained for CD4, CD25, CD127 and FoxP3 to stain for T regulatory cells as for DCs. The supernatant was collected was assayed for TGF-β, IFN-γ, IL-10 by ELISA.

Experiments were also performed where platelets were cultured with activated T cells. anti-CD3 and anti-CD28 coated Dynabeads (Invitrogen, CA) were used for T cell activation [45]. For proliferation measurements CFSE labeled T cells were used.

### Podoplanin staining on CD4 T cells

PBMCs were stained with CD4, CD45RA and CCR7 antibodies to identify naïve (CD4^+^CD45RA^+^CCR7^+^), central memory (CD4^+^CD45RA^−^CCR7^+^) and effector memory (CD4^+^CD45RA^−^CCR7^−^) subsets. Podoplanin was stained using a specific antibody from Biolegend, San Diego, CA.

### T reg induction by recombinant CLEC-2 protein and podoplanin crosslinking

Purified T cells (unactivated and activated as described above were cultured with recombinant CLEC-2 protein (RnD systems) at 1μg/ml for 96h. T cells were stained for T regs as described. Supernatants collected were assayed for IL-10, IFN-γ and TGF-β.

Purified T cells were cultured with Podoplanin antibody (1μg/ml) or isotype control for 1 h and subsequently crosslinked with secondary anti-rat antibody. 96h later T cells were stained for T regs as described. Supernatants collected were assayed for IL-10, IFN-γ and TGF-β.

### Statistical analysis

Statistical analysis was performed using Graph Pad Prism. Within group differences between unstimulated and stimulated conditions were tested using paired t-tests. Values of *p* < 0.05 were considered significant.
